# Pure *Trans*-Resveratrol Nanoparticles Prepared by a Supercritical Antisolvent Process Using Alcohol and Dichloromethane Mixtures: Effect of Particle Size on Dissolution and Bioavailability in Rats

**DOI:** 10.3390/antiox9040342

**Published:** 2020-04-22

**Authors:** Eun-Sol Ha, Heejun Park, Seon-Kwang Lee, Woo-Yong Sim, Ji-Su Jeong, In-hwan Baek, Min-Soo Kim

**Affiliations:** 1 College of Pharmacy, Pusan National University, 63 Busandaehak-ro, Geumjeong-gu, Busan 46241, Korea; edel@pusan.ac.kr (E.-S.H.); pharmacy4336@pusan.ac.kr (H.P.); lsk7079@pusan.ac.kr (S.-K.L.); popo923@pusan.ac.kr (W.-Y.S.); sui15@pusan.ac.kr (J.-S.J.); 2 College of Pharmacy, Kyungsung University, 309, Suyeong-ro, Nam-gu, Busan 48434, Korea; baek@ks.ac.kr

**Keywords:** resveratrol, nanoparticle, supercritical fluid, dissolution, bioavailability

## Abstract

The aim of this study was to prepare pure *trans*-resveratrol nanoparticles without additives (surfactants, polymers, and sugars) using a supercritical antisolvent (SAS) process with alcohol (methanol or ethanol) and dichloromethane mixtures. In addition, in order to investigate the effect of particle size on the dissolution and oral bioavailability of the *trans*-resveratrol, two microparticles with different sizes (1.94 μm and 18.75 μm) were prepared using two different milling processes, and compared to *trans*-resveratrol nanoparticles prepared by the SAS process. The solid-state properties of pure *trans*-resveratrol particles were characterized. By increasing the percentage of dichloromethane in the solvent mixtures, the mean particle size of *trans*-resveratrol was decreased, whereas its specific surface area was increased. The particle size could thus be controlled by solvent composition. *Trans*-resveratrol nanoparticle with a mean particle size of 0.17 μm was prepared by the SAS process using the ethanol/dichloromethane mixture at a ratio of 25/75 (*w*/*w*). The in vitro dissolution rate of *trans*-resveratrol in fasted state-simulated gastric fluid was significantly improved by the reduction of particle size, resulting in enhanced oral bioavailability in rats. The absolute bioavailability of *trans*-resveratrol nanoparticles was 25.2%. The maximum plasma concentration values were well correlated with the in vitro dissolution rate. These findings clearly indicate that the oral bioavailability of *trans*-resveratrol can be enhanced by preparing pure *trans*-resveratrol nanoparticles without additives (surfactants, polymers, and sugars) by the SAS process. These pure *trans*-resveratrol nanoparticles can be applied as an active ingredient for the development of health supplements, pharmaceutical products, and cosmetic products.

## 1. Introduction

*Trans*-resveratrol, a phytochemical with strong antioxidant property, is naturally found in grapes, berries, and peanuts. The benefits of *trans*-resveratrol have been reported in various diseases, including cancer, cardiovascular diseases, diabetes mellitus, neurological diseases, obesity, and other conditions associated with oxidative stress and inflammation [1,2,3,4,5]. Chemically, *trans*-resveratrol is converted into *cis*-resveratrol by light and has a very low aqueous solubility [6,7]. Further, its benefits are affected after its oral administration owing to its poor solubility and instability in physiological conditions as well as extensive metabolism [8,9]. To date, several formulation strategies such as amorphous solid dispersions, composite nanoparticles, emulsions, liposomes, polymeric micelles, self-emulsifying drug delivery systems, and solid lipid nanoparticles have been investigated to resolve this problem [10,11,12,13,14,15,16,17,18,19]. In addition, the enhanced oral bioavailability in humans has been reported with lipid formulation, micellar liquid formulation, and microparticles mixed with a liquid formulation [20,21,22]. Previously, the area under the plasma concentration versus time curve (AUC) and maximum plasma concentration (C_max_) values obtained after oral administration at a dose of 40-mg *trans*-resveratrol were approximately 10-fold and 8.5-fold higher in the capsule containing solubilized resveratrol in polysorbate 20 and polyglyceryl-3-dioleate mixture than in powder capsule (original powder), respectively [20]. In a case study using capsules containing solubilized resveratrol in polysorbate 80, polysorbate 20, and medium chain triacylglycerol mixtures, the intake of a liquid micellar formulation containing 30 mg *trans*-resveratrol produced higher AUC (5.0-fold increase) and C_max_ (10.6-fold increase) than the equivalent dose of the original powder [21]. In a human pharmacokinetic study using a suspension prepared by mixing resveratrol powder and the liquid formulation (docusate sodium solution and distilled water) at a daily dose of 5.0 g *trans*-resveratrol, the oral bioavailability of the micronized powder (particle size less than 5 μm) was significantly higher than that of the nonmicronized powder, with approximately 3.6-fold increases in the C_max_ values at 14 days [22]. However, as a health supplement, a large amount of *trans*-resveratrol must be administered for long periods. Minimizing the use of additives such as surfactants (polysorbates and docusate sodium) can reduce its side effects.

Particle size reduction for poorly water-soluble phytochemicals is an important process for increasing the dissolution rate and, consequently, the oral bioavailability of compounds [23,24,25,26]. Generally, nanoparticles can be prepared via two different approaches, the ‘top-down’ and ‘bottom-up’ approaches. In the ‘top-down’ and ‘bottom-up’ methods, water containing surface stabilizers was used as a dispersion medium or an antisolvent. To transform the phytochemicals into the solid form, nanosuspensions are dried by spray-drying and fluidized bed granulation after the addition of dispersants (usually sucrose and mannitol) to reduce the aggregation of nanoparticles during solidification and enhance nanoparticle redispersion in water [27,28]. These methods have some disadvantages, such as the use of massive additives. Alternative technologies, such as the supercritical fluid technology, can produce solid nanoparticles in a one-step process without the use of additives and a subsequent water removal process [29,30,31]. Therefore, we attempted to prepare pure *trans*-resveratrol nanoparticles without the additives (surfactants, polymers, and sugars) by using the supercritical antisolvent (SAS) process and alcohol and dichloromethane mixtures. Herein, the solid-state properties of the pure *trans*-resveratrol nanoparticles were characterized. Further, particle size reduction of *trans*-resveratrol was performed using a Fitz mill and an air jet-mill, and a dissolution and pharmacokinetic study was conducted to investigate the effect of particle size on the dissolution and oral bioavailability of *trans*-resveratrol.

## 2. Materials and Methods

### 2.1. Materials

*Trans*-resveratrol was purchased from Ningbo Liwah Pharmaceutical Co., Ltd. (Ningbo, China). United States Pharmacopeia (USP) reference standard of *trans*-resveratrol, dimethyl sulfoxide (DMSO), and polyethylene glycol (PEG) 300 were purchased from Sigma-Aldrich Co. (St Louis, MO, USA). Methanol, ethanol, and dichloromethane of high-performance liquid chromatography (HPLC) grade were purchased from Honeywell Burdick and Jackson (Muskegon, MI, USA). Carbon dioxide (99.99% purity) was supplied from Hana Gas Co., Ltd. (Gimhae, Korea).

### 2.2. Solubility Study of Trans-Resveratrol in the Alcohol and Dichloromethane Mixtures

To understand the solubility behavior of *trans*-resveratrol in the solvents, the solubility of *trans*-resveratrol in alcohol (methanol or ethanol) and dichloromethane mixtures was determined by the shake-flask method through solid–liquid equilibrium [32,33,34]. Briefly, 15 g of the solvent mixture was prepared in an amber vial by mixing alcohol (methanol or ethanol) and dichloromethane at different weight ratios (alcohol:dichloromethane; 0:100, 25:75, 50:50, 75:25, 100:0 *w*/*w*). Excess *trans*-resveratrol (raw material) was mixed with the solvents in amber vials via vortex for 15 min and sonication in an ultrasonic bath (5800 model, Branson, USA) for 45 min. The amber vials were shaken at 60 rpm in a water-bath mechanical shaker (BS-21, Jeiotech Co., Ltd., Daejeon, Korea) at 25 °C for at least 24 h. For HPLC analysis, the sample was filtered using a 0.2 μm syringe filter to obtain a clear supernatant from a saturated solution. Thereafter, the filtrate was placed in a preweighed volumetric flask. The concentration of *trans*-resveratrol was determined using a Prominence HPLC-ultraviolet (HPLC-UV) system (Shimadzu, Tokyo, Japan) after dilution with methanol. *Trans*-resveratrol was separated by Gemini C18 column (150 × 4.6 mm, 5 μm, Phenomenex, Torrance, CA, USA). The mobile phase consisted of acetonitrile/water (40:60, *v*/*v*). The flow rate was 0.8 mL/min, column temperature was 30 °C, and the detection wavelength was 303 nm. All solubility measurements were repeated three times.

### 2.3. Preparation of Pure Trans-Resveratrol Nanoparticles Using the SAS Process

Pure *trans*-resveratrol nanoparticles were prepared by the SAS process, as previously reported [35,36,37]. To prepare the sample solution, raw *trans*-resveratrol (30 mg/g) was dissolved in alcohol (methanol or ethanol) and dichloromethane at different weight ratios (alcohol: dichloromethane; 25:75, 50:50, 75:25, 100:0 *w*/*w*) in an amber vial. Carbon dioxide liquefied with a cooler was heated to a desired point (40 °C) using a heat exchanger, and then injected into the particle formation vessel (1 L) through a two-way nozzle using a syringe pump (ISCO 260D dual-pump continuous flow systems, Teledyne Technologies Inc., Thousand Oaks, CA, USA). Once a pressure of 150 bar and temperature of 40 °C were achieved and equilibrium was reached, supercritical carbon dioxide was delivered at a rate of 40 g/min and the sample solution was simultaneously injected at a rate of 0.5 g/min using an HPLC pump (ReaXus LD Class, Teledyne Technologies Inc., Thousand Oaks, CA, USA). Upon injecting the sample solution, only carbon dioxide was further pumped at the same rate to remove the residual solvent that could dissolve the prepared particles. The SAS system was slowly depressurized to atmospheric pressure. The generated particles were collected on the walls and bottom of the particle formation vessel.

### 2.4. Particle Size Reduction of Trans-Resveratrol Using Fitz Mill or Air Jet-Mill

Two different milling processes using a Fitz mill and an air jet-mill were applied for particle size reduction of raw *trans*-resveratrol. To generate *trans*-resveratrol microparticles with the mean particle size of approximately 20 μm, raw *trans*-resveratrol with a mean particle size of 108.94 μm was milled in the Fitz mill (Fitz Mill L1A, Fitzpatrick Company, Westwood, MA, USA) under the following conditions: speed, 500–1000 rpm; feed rate, 0.5 g/min. To obtain *trans*-resveratrol microparticles with a mean particle size of approximately 2 μm, continuous grinding using an air jet-mill (McOne, Jetpharma, Balerna, Switzerland) was conducted under the following process conditions: injection pressure, 8 bar; grinding pressure, 8 bar; and feed rate, 0.5 g/min.

### 2.5. Analysis of Trans-Resveratrol Purity and Residual Solvent

The purity of *trans*-resveratrol after the SAS process and milling process was validated by HPLC analysis. The sample solution and standard solution were prepared by dissolving the processed *trans*-resveratrol and USP reference standard in methanol at 100 μg/mL concentration, respectively. A 10-μL volume of the solution was injected into a Prominence HPLC-ultraviolet (HPLC-UV) system (Shimadzu, Tokyo, Japan) as described above. Residual solvents of *trans*-resveratrol after the SAS process were measured by a gas chromatograph (GC) (Hewlett-Packard, 5890 SERIES II, Palo Alto, CA, USA) coupled with head space sampler (Hewlett-Packard, 7694 headspace sampler, Palo Alto, CA, USA) and flame ionization detector. Analysis was performed as previously described with slight modifications [38]. The fused-silica capillary column (Phenomenex, model ZB-624, 30 m length × 0.32 mm I.D. (internal diameter), Torrence, CA, USA) was selected for the simultaneous separation of ethanol, methanol, and dichloromethane in the sample solution. The following programmed GC conditions were employed: the initial temperature of 35 °C was held for 10 min after injection, then increased at a rate of 15 °C/min to 40 °C, where the temperature maintained for 10 min, then increased at a rate of 18 °C/min to 235 °C. After holding for 8 min at 235 °C, the temperature was returned to its initial value. The headspace conditions were: equilibration time, 60 min at 80 °C with gentle shaking; pressurization time, 0.5 min; and loop fill time, 0.1 min. The test sample solution was prepared by dissolving 100 mg of each sample dissolved in 10 mL of DMSO in 10 mL vials. Analyses were performed in triplicate for each sample.

### 2.6. Solid-State Characterizations

#### 2.6.1. Scanning Electron Microscopy

The morphology of the *trans*-resveratrol nanoparticles and microparticles was observed using a scanning electron microscope (SUPRA 25 or 40, Zeiss, Oberkochen, Germany). Electrically conductive samples were prepared by gold coating in vacuum for 1 min, before observation. Samples were visualized at an accelerating voltage of 5 kV.

#### 2.6.2. Particle Size Measurements

For the nanoparticles, the z-average size and polydispersity index (PI) were measured using ELSZ-1000 (Otsuka Electronics, Tokyo, Japan) according to the dynamic light scattering (DLS) method. In addition, particle size and size distribution of *trans*-resveratrol nanoparticles and microparticles were determined using laser diffraction (LS 13 320, Coulter Beckman, Brea, CA, USA) according to the Mie scattering theory. Samples were sufficiently dispersed in water, and all particle size measurements were repeated three times.

#### 2.6.3. Specific Surface Area Measurements

The specific surface area of *trans*-resveratrol nanoparticles and microparticles was determined using the Micromeritics TriStar II 3020 instrument (Micromeritics, Norcross, GA, USA) based on the Brunauer–Emmett–Teller method with the nitrogen adsorption analysis after degassing samples with helium (purity > 99.99%).

#### 2.6.4. Differential Scanning Calorimetry (DSC) Analysis

DSC measurements were conducted with a Discovery DSC 25 instrument connected to an RSC90 cooling system (TA Instruments, Inc., New Castle, DE, USA). High purity indium and aluminum oxide sapphire were used to calibrate temperature and heat capacity. The *trans*-resveratrol samples ranging from 3 to 4 mg were accurately weighed and sealed in an aluminum pan. The same hermetic aluminum pan without sample was used as a reference. The measurements were carried out under nitrogen purge (300 mL/min) in the temperature range of 0 to 300 °C, with a heating rate of 10 °C/min. All measurements for each sample were performed in triplicate.

#### 2.6.5. Powder X-Ray Diffraction (PXRD) Analysis

PXRD analysis was carried out using an X-ray diffractometer (XPert 3, Panalytical, Almelo, Netherlands), with Cu-Kα radiation at a voltage of 40 kV and a current of 40 mA. The samples were scanned from 5° to 50° (2θ), with a scanning speed of 3°/min and a step size of 0.01°.

### 2.7. Dissolution Study

Dissolution under the physiological conditions of the gastrointestinal tract is the first process in the oral absorption of *trans*-resveratrol. To investigate the effect of particle size on dissolution and to derive the relationship between in vitro dissolution rate and in vivo pharmacokinetic parameters, we conducted dissolution testing of *trans*-resveratrol nanoparticles and microparticles in fasted state-simulated gastric fluid (FaSSGF) and fasted state-simulated intestinal fluid (FaSSIF) as biorelevant dissolution media. First, the solubility of *trans*-resveratrol (raw material) in FaSSGF and FaSSIF was determined at 37 °C as described in the solubility measurement method above. FaSSGF and FaSSIF were prepared with sodium chloride, hydrochloric acid, and Biorelevant powder (Biorelevant.com Ltd., London, UK) and sodium hydroxide, sodium phosphate monobasic, sodium chloride, and Biorelevant powder, respectively, according to the method retrieved from Biorelevant.com. The dissolution test was carried out in accordance with the USP paddle method using a Varian VK 7010 dissolution system (Vankel Industries, Edison, NJ, USA) at 50 rpm and 37 ± 0.5 °C. To neglect the effect of wettability, *trans*-resveratrol nanoparticles and microparticles were dispersed in water at 50 mg/mL prior to the dissolution study. A sample of 50 mg *trans*-resveratrol was placed in 900 mL of dissolution media (FaSSGF). Dissolved *trans*-resveratrol was quantified using Pion Rainbow spectrometers with 2-mm path length UV fiber optic probes (Pion Inc., Billerica, MA, USA) at a wavelength of 306 nm. Data were collected every 1 min for 60 min, and all dissolution measurements were repeated six times.

### 2.8. Pharmacokinetic Study in Rats

To investigate the effect of particle size on bioavailability, the pharmacokinetic parameters of *trans*-resveratrol nanoparticles and microparticles were obtained following their oral administration to male Sprague–Dawley (SD) rats. The animal study protocol complied with the institutional guidelines for the care and use of laboratory animals and was approved by the ethics committee of Kyungsung University (No. 19-008A). Twenty-four male SD rats (200–230 g; HanaBiotech, Suwon, Korea) were divided into four treatment groups (*n* = 6 per group). Three groups received either *trans*-resveratrol nanoparticles (0.17 μm) prepared by the SAS process, *trans*-resveratrol microparticles (1.94 μm) milled by the air jet-mill, and *trans*-resveratrol microparticles (18.25 μm) milled by the Fitz mill at a dose of 50 mg/kg using an oral dosing apparatus. Each particle was dispersed in 1 mL of water immediately before dosing [39]. To estimate the bioavailability (F%) of *trans*-resveratrol nanoparticles and microparticles, one group was intravenously administered via the tail vein a dose of 15 mg/2.5 mL/kg (solution prepared by dissolving *trans*-resveratrol in DMSO and PEG 300 mixtures, 15:85 *v*/*v*) [40]. Serial blood samples (approximately 300 μL each) were collected from the jugular vein at different time intervals. The blood samples were centrifuged (12,000 rpm, 5 min), and plasma was collected and stored at −70 °C until HPLC analysis. The concentration of *trans*-resveratrol in plasma was determined using a previously reported method and under HPLC analysis conditions [16,41]. The AUC_0→8h_ was calculated using the linear trapezoidal method. C_max_ and the time required to reach *C*_max_ (*T*_max_) were directly obtained from plasma data.

### 2.9. Data Analysis

Data are expressed as mean ± standard deviation (*n* = 3, 4, or 6). One-way analysis of variance followed by the least significant difference test and the Student–Newman–Keuls test was performed using SPSS (version 25.0; IBM SPSS Statistics, IBM Corporation, Armonk, NY, USA).

## 3. Results and Discussion

### 3.1. Pure Trans-Resveratrol Nanoparticles Prepared by the SAS Process

**Figure 1:**
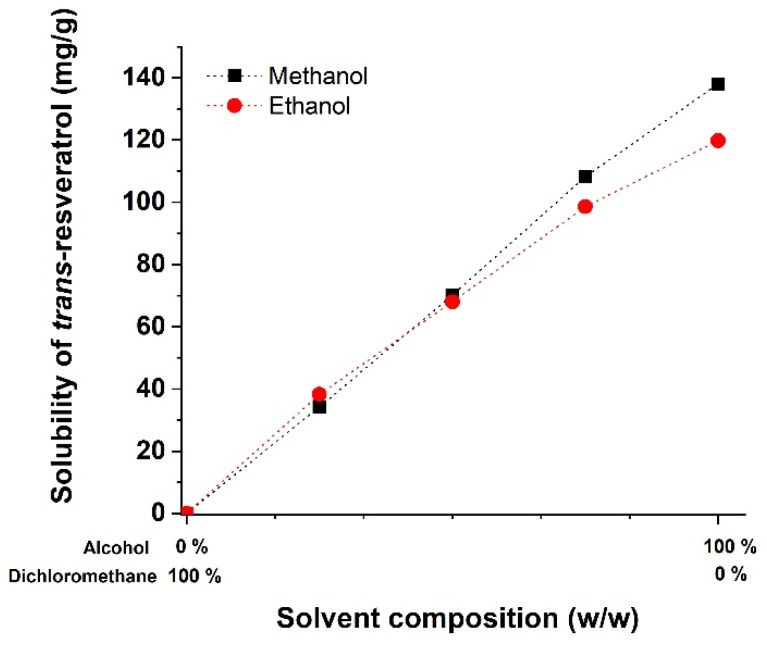
Solubility of *trans*-resveratrol in different alcohol–dichloromethane mixtures at 25 °C.

To prepare the *trans*-resveratrol nanoparticles using the SAS process, the solubility of *trans*-resveratrol was determined in alcohol (methanol or ethanol) and dichloromethane mixtures at different weight ratios (alcohol: dichloromethane; 0:100, 25:75, 50:50, 75:25, 100:0 *w*/*w*) and the measured data were graphically plotted (Figure 1). The solubility of *trans*-resveratrol in dichloromethane was 5.38 μg/mg, indicating very low solubility. However, the solubility of *trans*-resveratrol was markedly increased by the addition of a small amount of alcohol, and reached 38.26 mg/g in the ethanol and dichloromethane mixture at 25:75 (*w*/*w*). As shown in Figure 1, solubility was proportionally increased with an increase in the alcohol ratio with mixtures of methanol and ethanol. Based on solubility, dichloromethane and alcohol (methanol and ethanol) were recognized as “poor solvent” and “good solvent,” respectively, with respect to *trans*-resveratrol.

**Figure 2:**
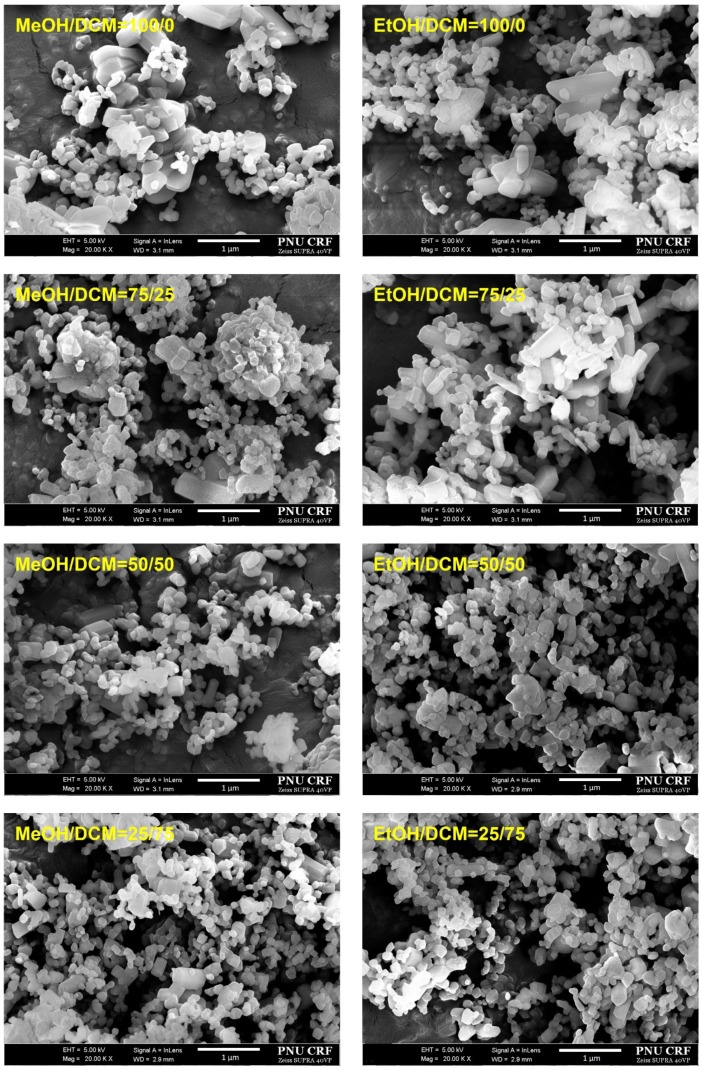
Scanning electron microscopy images of the *trans*-resveratrol nanoparticles prepared by the SAS process using different alcohol–dichloromethane mixtures. MeOH: methanol, DCM: dichloromethane, EtOH: ethanol.

Based on the solubility data, we prepared *trans*-resveratrol nanoparticles using the SAS process and 30 mg/g of *trans*-resveratrol to investigate the effect of solvent composition (alcohol: dichloromethane; 25:75, 50:50, 75:25, 100:0 *w*/*w*) on particle size and morphology. As shown in Figure 2 and Table 1, the *trans*-resveratrol precipitated from the methanol–dichloromethane mixtures had smaller nanoparticles than those generated from methanol. By increasing the percentage of dichloromethane in the solvents, the mean particle size decreased and the specific surface area increased. The PI values for *trans*-resveratrol precipitated from the methanol/dichloromethane mixtures at ratios 25:75 and 50:50 (*w*/*w*) were below 0.2, indicating narrow particle size distribution [42,43]. Cohesion of the particles was observed, which may be due to the relatively high surface energy of fine nanoparticles. From the results obtained, nanoparticles were easily dispersed in water, and the particle sizes measured by DLS method were in good agreement with the SEM images, and it was confirmed that the particles were not aggregated via irreversible bonding. In most particle formation studies using the SAS process, a single solvent is employed to dissolve compounds. By performing some case studies using solvent mixtures, De Marco et al. [44,45] reported that the particle size and morphology of compounds were controlled by the solvent mixtures with a “poor solvent” and “good solvent” through the enhanced mixing effect of the “poor solvent.” When ethanol and the ethanol–dichloromethane mixtures were used, a similar effect to that of dichloromethane in the methanol–dichloromethane mixtures was observed as the particle size of *trans*-resveratrol was reduced. In particular, the *trans*-resveratrol nanoparticles prepared at a ratio of 25:75 (ethanol:dichloromethane) had a mean particle size of 151.2 nm, with 60.23 m^2^/g. Additionally, it had the smallest particle size among the prepared samples.

**Figure 3:**
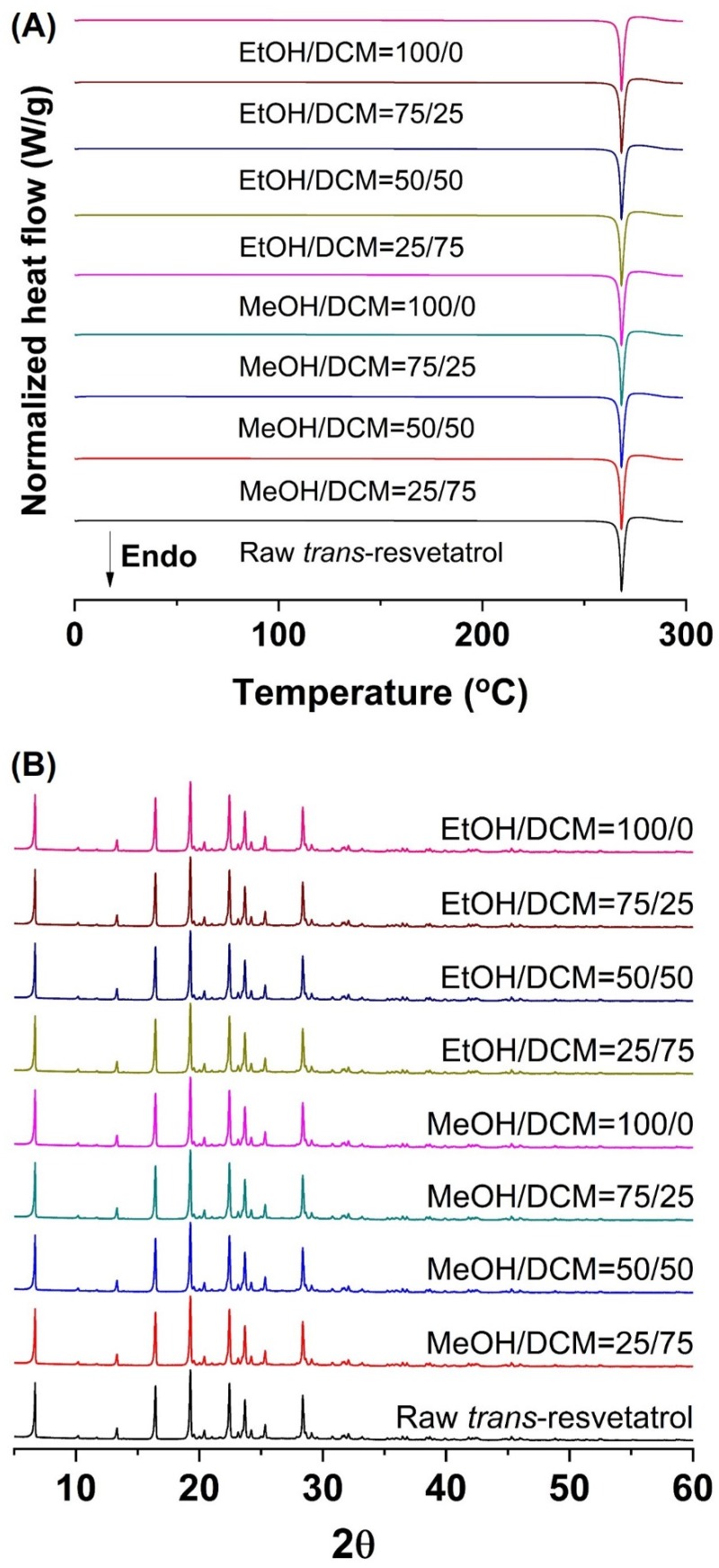
Differential scanning calorimetry thermograms (**A**) and powder X-ray diffraction patterns (**B**) of the *trans*-resveratrol nanoparticles prepared by the SAS process using different alcohol–dichloromethane mixtures. MeOH: methanol, DCM: dichloromethane, EtOH: ethanol.

As shown in Figure 3 and Table S1 (Supplementary Material), the melting temperature, fusion enthalpy, and X-ray characteristic peaks of *trans*-resveratrol in all processed samples were the same as those of the unprocessed raw *trans*-resveratrol (*p* > 0.05), which means that there was no difference in crystal polymorphism and crystallinity between nanoparticle samples prepared by the SAS process. The process yield was above 85% for all experiments. All *trans*-resveratrol nanoparticles prepared using the SAS process exhibited low residual solvents; methanol < 20 ppm, ethanol < 20 ppm, and dichloromethane < 10 ppm according to the residual solvent analysis. The purity (%) of all *trans*-resveratrol nanoparticles was above 99.3%, indicating no degradation and conversion to *cis*-resveratrol during the SAS process. Therefore, pure *trans*-resveratrol nanoparticles were successfully prepared using the SAS process with alcohol and dichloromethane and their particle size could be controlled by solvent composition. Based on the above results, *trans*-resveratrol nanoparticles prepared at a ratio of 25:75 (ethanol:dichloromethane, *w*/*w*) were selected as the best sample for further investigation.

### 3.2. Effect of Particle Size on Dissolution and Oral Bioavailability

To investigate the effect of particle size on dissolution and oral bioavailability, two different sizes of *trans*-resveratrol microparticles were prepared using a Fitz mill and an air jet-mill in contrast to the nanoparticles prepared by the SAS process. As a result, the particle size of raw *trans*-resveratrol with a mean particle size of 108.94 μm was decreased to 18.25 μm by the milling process using the Fitz mill and 1.94 μm by the air jet-mill. In contrast, the *trans*-resveratrol nanoparticles with mean particle sizes of 0.17 μm and 60.23 m^2^/g were prepared by the SAS process using the ethanol: dichloromethane mixture at a ratio of 25:75. The physicochemical properties of the *trans*-resveratrol nanoparticles and microparticles are summarized in Table 2. The melting temperature and fusion enthalpy of *trans*-resveratrol nanoparticles and microparticles were not significantly different (*p* > 0.05) to those of the unprocessed raw *trans*-resveratrol (Table S1, Supplementary material). The results of DSC and PXRD confirmed that there was no difference in crystal polymorphism and crystallinity between nanoparticles and microparticles.

**Figure 4:**
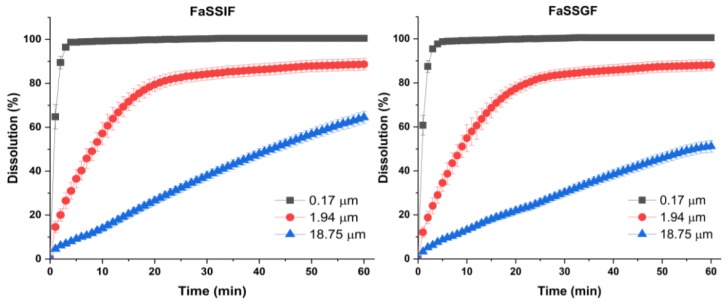
Effect of particle size on the dissolution of *trans*-resveratrol in the fasted state-simulated gastric fluid (FaSSGF) and fasted state simulated intestinal fluid (FaSSIF).

To investigate the effect of particle size on dissolution and derive the relationship between in vitro dissolution rate and in vivo pharmacokinetic parameters, we carried out a dissolution test with the *trans*-resveratrol nanoparticles and microparticles in FaSSGF and FaSSIF as biorelevant dissolution media. To date, the solubility of *trans*-resveratrol in these dissolution media has not been reported in the literature. Based on this study, the solubility values of *trans*-resveratrol in FaSSGF and FaSSIF at 37 °C were 61.59 ± 1.01 μg/mL and 87.15 ± 1.31 μg/mL, respectively. The solubility in FaSSIF (pH 6.5) was superior to that in FaSSGF (pH 1.5) and could be attributed to micellar solubilization instead of pH as a result of the higher concentration of sodium taurocholate and lecithin; this is because *trans*-resveratrol exhibited a similar solubility in the pH range of 1.2–6.8 [10,46]. To predict the in vivo dissolution and investigate the effect of particle size, 50 mg of *trans*-resveratrol was applied for solubilization in 900 mL of FaSSGF or FaSSIF. The dissolution profiles of the *trans*-resveratrol nanoparticles and microparticles in FaSSGF and FaSSIF are shown in Figure 4. Evidently, the dissolution rate of *trans*-resveratrol was affected by its particle size in both FaSSGF and FaSSIF. As a nanoparticle (0.17 μm), *trans*-resveratrol was approximately 90% dissolved within 3 min; however, as a microparticle (18.25 μm), only ~50% dissolved at 60 min in both FaSSGF and FaSSIF. This enhancement in the dissolution rate is a consequence of the increase in surface area and decrease in diffusion layer thickness by particle size reduction. For further quantitative analysis, the Hixson–Crowell cube root equation was applied to estimate the resveratrol release rate and simulated 50% dissolution time (*t*_50%_) [47]. As shown in Table 3, the resveratrol release rate and simulated 50% dissolution time (*t*_50%_) for the *trans*-resveratrol nanoparticles and microparticles were increased and decreased, respectively, as the particle size was reduced.

**Figure 5:**
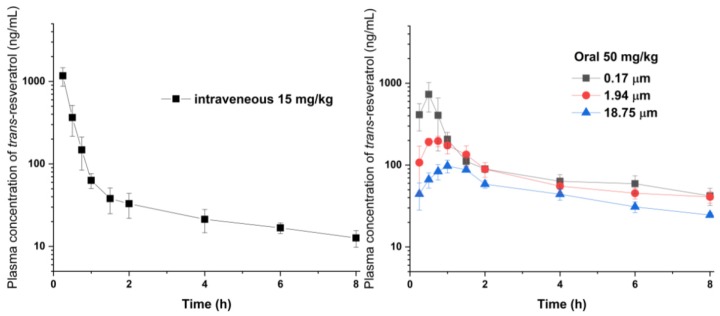
Effect of particle size on the plasma concentration of *trans*-resveratrol after oral administration to Sprague–Dawley (SD) rats. Data are expressed as mean ± standard deviation (*n* = 6).

To investigate the effect of particle size on the bioavailability, the pharmacokinetic parameters of *trans*-resveratrol nanoparticles and microparticles were obtained following their oral administration to male SD rats (Table 4). In addition, the absolute oral bioavailability (F%) of the *trans*-resveratrol nanoparticles and microparticles were estimated using the mean value of the intravenously administered AUC_0→8 h_. As shown in Figure 5, the nanoparticles achieved C_max_ within 0.5 h after oral administration and had a higher concentration than the microparticles. For the pure *trans*-resveratrol particles lacking solubilizing additives, the rapid dissolution generated by the 0.17 μm nanoparticles led to rapid oral absorption, resulting in enhanced C_max_. However, the degree of enhancement via particle size reduction was relatively small for the AUC values, which may be due to the rapid metabolism of resveratrol [48]. Nonetheless, the absolute bioavailability of pure *trans*-resveratrol nanoparticles prepared using the SAS process was 25.2%. Previously, the absolute bioavailability of *trans*-resveratrol was reported to exist between 2.6% and 46.4%, thereby varying according to the sample preparation (the type and amount of suspending agent and surfactant) employed for oral administration in rats [38,39,49,50,51].

**Figure 6:**
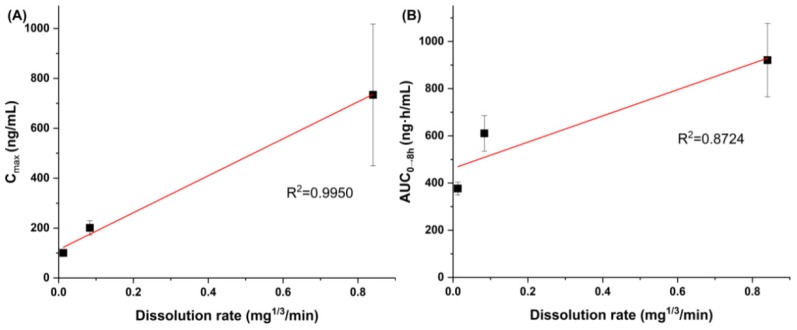
Correlation between the in vitro dissolution rate and in vivo pharmacokinetic data of *trans*-resveratrol: (**A**) in vitro dissolution rate vs. in vivo C_max_; (**B**) in vitro dissolution rate vs. in vivo AUC_0–8 h_.

After oral administration, dissolution in the physiological condition of the stomach is the first process in the oral absorption of *trans*-resveratrol. Thus, the dissolution data obtained in FaSSGF were employed to establish the relationship between in vitro dissolution rate and in vivo pharmacokinetic parameters (Figure 6). C_max_ values were well correlated with the in vitro dissolution rate compared with the AUC_0→8 h_ values. In fact, the in vitro dissolution rate controlled by particle size could predict the in vivo pharmacokinetic parameters for *trans*-resveratrol.

## 4. Conclusions

Pure *trans*-resveratrol nanoparticles can be successfully prepared using the SAS process with alcohol (methanol or ethanol) and dichloromethane. We demonstrated that their particle size is controlled by solvent composition. Further, we revealed that the in vitro dissolution rate of *trans*-resveratrol is significantly improved by a reduction in particle size, thereby enhancing the oral bioavailability. These findings clearly indicate that the oral bioavailability of *trans*-resveratrol can be enhanced by preparing pure *trans*-resveratrol nanoparticles without additives (surfactants, polymers, and sugars) by the SAS process. This pure *trans*-resveratrol nanoparticle can be used as an active ingredient in the development of health supplements, pharmaceutical products, and cosmetic products.

## Figures and Tables

**Table 1:** Particle size (dynamic light scattering method, DLS) and specific surface area of *trans*-resveratrol nanoparticles prepared by the SAS process using different alcohol–dichloromethane mixtures.

Solvent Composition Mass%	Z-Average (nm)	PI	Specific Surface Area (m^2^/g)
MeOH/DCM = 25/75	174.5 ± 8.5	0.182 ± 0.029	56.18 ± 0.88
MeOH/DCM = 50/50	208.5 ± 10.5	0.195 ± 0.039	45.22 ± 0.63
MeOH/DCM = 75/25	393.3 ± 13.3	0.305 ± 0.079	31.14 ± 0.55
MeOH/DCM = 100/0	501.7 ± 15.1	0.311 ± 0.081	27.51 ± 0.83
EtOH/DCM = 25/75	151.2 ± 5.9	0.174 ± 0.013	60.23 ± 0.98
EtOH/DCM = 50/50	194.2 ± 9.9	0.189 ± 0.023	49.31 ± 0.53
EtOH/DCM = 75/25	371.8 ± 12.9	0.285 ± 0.049	35.29 ± 0.65
EtOH/DCM = 100/0	481.5 ± 12.5	0.291 ± 0.061	29.22 ± 0.45

Data are expressed as mean ± standard deviation (*n* = 3). PI: polydispersity index, MeOH: methanol, DCM: dichloromethane, EtOH: ethanol. PI was used to indicate the width of particle size distribution. A low PI (usually less than 0.2) indicates a monodispersed sample.

**Table 2:** Physicochemical properties of *trans*-resveratrol nanoparticles and microparticles used to investigate the effect of particle size on the dissolution and oral bioavailability

Characterization	Nanoparticles	Microparticles	Microparticles
Preparation method and condition	SAS process: 40 °C and 150 bar, Solvent composition = 25:75 ethanol:dichloromethane (*w*/*w*)	Air jet-milling: Injection pressure = 8 bar, Grinding pressure = 8 bar, Feed rate = 0.5 g/min	Fitz milling: Screw speed = 500–1000 rpm and feed rate = 0.5 g/min
Morphology (scanning electron microscopy)	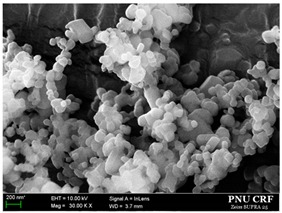	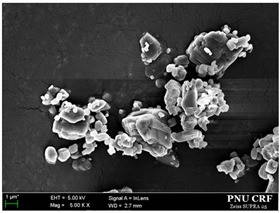	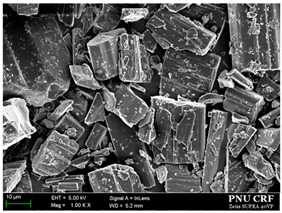
DSC	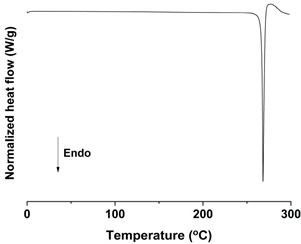	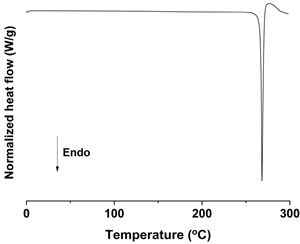	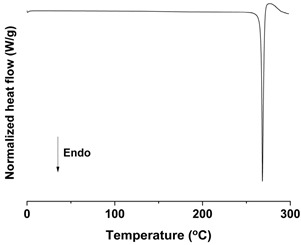
PXRD	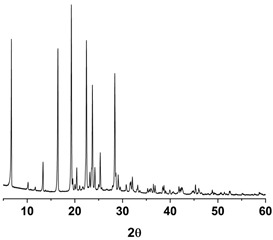	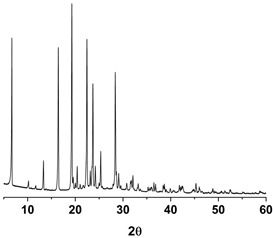	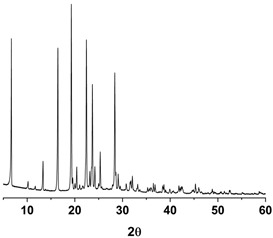
Mean particle size (μm)	0.17 ± 0.05	1.94 ± 0.26	18.75 ± 0.53
*d*_10_ (μm)	0.09	0.95	5.23
*d*_50_ (μm)	0.19	2.12	19.23
*d*_90_ (μm)	0.33	5.23	45.39
Span	1.26	2.02	2.14
Specific surface area (m^2^/g)	60.23 ± 0.98	3.43 ± 0.09	0.31 ± 0.03
Purity (%)	99.4	99.2	99.3

For comparison, the particle size and size distribution of the *trans*-resveratrol nanoparticles and microparticles were determined using the laser diffraction method. Span = (*d*_90_−*d*_10_)/*d*_50_, where *d*_10_, *d*_50_, and *d*_90_ are the diameters and the given percentage value is the percentage of particles smaller than that size. DSC: differential scanning calorimetry, PXRD: powder X-ray diffractometry.

**Table 3:** Dissolution rate and simulated 50% dissolution time of the *trans*-resveratrol nanoparticles and microparticles determined using the Hixson–Crowell equation

Particle Size	Dissolution Rate, *k* (mg^1/3^/min)		Simulated 50% Dissolution Time, *t*_50%_ (mins)	
	FaSSGF	FaSSIF	FaSSGF	FaSSIF
0.17 μm	0.8404	0.8843	0.9	0.9
1.94 μm	0.0836	0.0894	9.1	8.5
18.75 μm	0.0128	0.0177	59.4	42.9

Hixson–Crowell equation: W_0_^1/3^ − W_t_^1/3^ = *kt*, W_0,_ the initial amount of resveratrol, W_t_, the remaining amount of resveratrol at time *t*, *k*, the resveratrol release rate. *t*_50%_: the time necessary for 50% resveratrol dissolution calculated using the Hixson–Crowell equation. FaSSGF: fasted state-simulated gastric fluid, FaSSIF: fasted state simulated intestinal fluid.

**Table 4:** Pharmacokinetic data of *trans*-resveratrol after oral administration and intravenous administration.

Particle Size, Route	AUC_0→8 h_ (ng·h/mL)	C_max_ (ng/mL)	T_max_ (h)	F (%)
0.17 μm, oral	920.4 ± 155.3 ^a,b^	734.0 ± 284.3 ^a,b^	0.5 ± 0.1	25.2
1.94 μm, oral	610.2 ± 75.5 ^a^	200.8 ± 28.6	0.8 ± 0.2	16.7
18.75 μm, oral	376.5 ± 27.4	100.2 ± 14.5	1.0 ± 0.3	10.3
Intravenous	1093.6 ± 301.5			

The absolute oral bioavailability (F%) was determined by dividing the mean value of orally administered AUC_0→8 h_ by the mean value of intravenously administered AUC_0→8 h_ with dose normalization. ^a^ *p* < 0.05 vs. 18.75 μm *trans*-resveratrol; ^b^ *p* < 0.05 vs. 1.94 μm *trans*-resveratrol. Data are expressed as mean ± standard deviation (*n* = 6). AUC_0→8 h_, the area under the plasma concentration versus time curve; C_max_, the maximum plasma concentration of *trans*-resveratrol; T_max_, the time required to reach C_max_.

## Supplementary Materials

The following are available online at https://www.mdpi.com/2076-3921/9/4/342/s1, Table S1: DSC data of raw material, SAS processed nanoarticles and milled microparticles consisted of pure trans-resveratrol.

## References

[1] Amri A., Chaumeil J.C., Sfar S., Charrueau C. (2012). Administration of resveratrol: What formulation solutions to bioavailability limitations?. J. Control. Release.

[2] Ramírez-Garza S.L., Laveriano-Santos E.P., Marhuenda-Muñoz M., Storniolo C.E., Tresserra-Rimbau A., Vallverdú-Queralt A., Lamuela-Raventós R.M. (2018). Health effects of resveratrol: Results from human intervention trials. Nutrients.

[3] Na J.-I., Shin J.-W., Choi H.-R., Kwon S.-H., Park K.-C. (2019). Resveratrol as a multifunctional topical hypopigmenting agent. Int. J. Mol. Sci..

[4] Fonseca J., Moradi F., Valente A.J.F., Stuart J.A. (2018). Oxygen and glucose levels in cell culture media determine resveratrol’s effects on growth, hydrogen peroxide production, and mitochondrial dynamics. Antioxidants.

[5] Chimento A., De Amicis F., Sirianni R., Sinicropi M.S., Puoci F., Casaburi I., Saturnino C., Pezzi V. (2019). Progress to improve oral bioavailability and beneficial effects of resveratrol. Int. J. Mol. Sci..

[6] Zhao Y., Shi M., Ye J.-H., Zheng X.-Q., Lu J.-L., Liang Y.-R. (2015). Photo-induced chemical reaction of trans-resveratrol. Food Chem..

[7] Marier J.F., Vachon P., Gritsas A., Zhang J., Moreau J.P., Ducharme M.P. (2002). Metabolism and disposition of resveratrol in rats: Extent of absorption, glucuronidation, and enterohepatic recirculation evidenced by a linked-rat model. J. Pharmacol. Exp. Ther..

[8] Berman A.Y., Motechin R.A., Wiesenfeld M.Y., Holz M.K. (2017). The therapeutic potential of resveratrol: A review of clinical trials. Npj Precis. Oncol..

[9] Walle T. (2011). Bioavailability of resveratrol. Ann. N. Y. Acad. Sci..

[10] Ha E.-S., Sim W.-Y., Lee S.-K., Jeong J.-S., Kim J.-S., Baek I.-H., Choi D.H., Park H., Hwang S.-J., Kim M.-S. (2019). Preparation and evaluation of resveratrol-loaded composite nanoparticles using a supercritical fluid technology for enhanced oral and skin delivery. Antioxidants.

[11] Mamadou G., Charrueau C., Dairou J., Nzouzi N.L., Eto B., Ponchel G. (2017). Increased intestinal permeation and modulation of presystemic metabolism of resveratrol formulated into self-emulsifying drug delivery systems. Int. J. Pharm..

[12] Intagliata S., Modica M.N., Santagati L.M., Montenegro L. (2019). Strategies to improve resveratrol systemic and topical bioavailability: An update. Antioxidants.

[13] Aguiar G.P.S., Boschetto D.L., Chaves L.M.P.C., Arcari B.D., Piato A.L., Oliveira J.V., Lanza M. (2016). Trans-resveratrol micronization by SEDS technique. Ind. Crops Prod..

[14] Zu Y., Zhang Y., Wang W., Zhao X., Han X., Wang K., Ge Y. (2016). Preparation and in vitro/in vivo evaluation of resveratrol-loaded carboxymethyl chitosan nanoparticles. Drug Deliv..

[15] Dhakar N.K., Matencio A., Caldera F., Argenziano M., Cavalli R., Dianzani C., Zanetti M., López-Nicolás J.M., Trotta F. (2019). Comparative evaluation of solubility, cytotoxicity and photostability studies of resveratrol and oxyresveratrol loaded nanosponges. Pharmaceutics.

[16] Kuk D.-H., Ha E.-S., Ha D.-H., Sim W.-Y., Lee S.-K., Jeong J.-S., Kim J.-S., Baek I.-H., Park H., Choi D.H. (2019). Development of a resveratrol nanosuspension using the antisolvent precipitation method without solvent removal, based on a quality by design (QbD) approach. Pharmaceutics.

[17] He H., Zhang Q., Wang J.-R., Mei X. (2017). Structure, physicochemical properties and pharmacokinetics of resveratrol and piperine coparticles. Cryst. Eng. Comm..

[18] Shimojo A.A.M., Fernandes A.R.V., Ferreira N.R.E., Sanchez-Lopez E., Santana M.H.A., Souto E.B. (2019). Evaluation of the influence of process parameters on the properties of resveratrol-loaded NLC using 2^2^ full factorial design. Antioxidants.

[19] Aguiar G.P.S., Arcari B.D., Chaves L.M.P.C., Magro C.D., Boschetto D.L., Piato A.L., Lanza M., Oliveira J.V. (2018). Micronization of trans-resveratrol by supercritical fluid: Dissolution, solubility and in vitro antioxidant activity. Ind. Crops Prod..

[20] Amiot M.J., Romier B., Dao T.M., Fanciullino R., Ciccolini J., Burcelin R., Pechere L., Emond C., Savouret J.F., Seree E. (2013). Optimization of trans-resveratrol bioavailability for human therapy. Biochimie.

[21] Calvo-Castro L.A., Schiborr C., David F., Ehrt H., Voggel J., Sus N., Behnam D., Bosy-Westphal A., Frank J. (2018). The oral bioavailability of trans-resveratrol from a grapevine-shoot extract in healthy humans is significantly increased by micellar solubilization. Mol. Nutr. Food Res..

[22] Howells L.M., Berry D.P., Elliott P.J., Jacobson E.W., Hoffmann E., Hegarty B., Brown K., Steward W.P., Gescher A.J. (2011). Phase I randomized, double-blind pilot study of micronized resveratrol (SRT501) in patients with hepatic metastases—Safety, pharmacokinetics, and pharmacodynamics. Cancer Prev. Res..

[23] Hajjar B., Zier K.-I., Khalid N., Azarmi S., Löbenberg R. (2018). Evaluation of a microemulsion-based gel formulation for topical drug delivery of diclofenac sodium. J. Pharm. Investig..

[24] Liu X., Feng X., Williams III R.O., Zhang F. (2018). Characterization of amorphous solid dispersions. J. Pharm. Investig..

[25] Ma X., Williams R.O. (2018). Polymeric nanomedicines for poorly soluble drugs in oral delivery systems: An update. J. Pharm. Investig..

[26] Singh D., Bedi N., Tiwary A.K. (2018). Enhancing solubility of poorly aqueous soluble drugs: Critical appraisal of techniques. J. Pharm. Investig..

[27] Gigliobianco M.R., Casadidio C., Censi R., Di Martino P. (2018). Nanoparticles of poorly soluble drugs: Drug bioavailability and physicochemical stability. Pharmaceutics.

[28] Ding Z., Wang L., Xing Y., Zhao Y., Wang Z., Han J. (2019). Enhanced oral bioavailability of celecoxib nanoparticleline solid dispersion based on wet media milling technique: Formulation, optimization and in vitro/in vivo evaluation. Pharmaceutics.

[29] Kim M.-S., Jin S.-J., Kim J.-S., Park H.J., Song H.S., Neubert R.H.H., Hwang S.-J. (2008). Preparation, characterization and in vivo evaluation of amorphous atorvastatin calcium nanoparticles using supercritical antisolvent (SAS) process. Eur. J. Pharm. Biopharm..

[30] Ha E.-S., Kim J.-S., Lee S.-K., Sim W.-Y., Jeong J.-S., Kim M.-S. (2019). Solubility and modeling of telmisartan in binary solvent mixtures of dichloromethane and (methanol, ethanol, n-propanol, or n-butanol) and its application to the preparation of nanoparticles using the supercritical antisolvent technique. J. Mol. Liq..

[31] Ha E.-S., Kim J.-S., Lee S.-K., Sim W.-Y., Jeong J.-S., Kim M.-S. (2019). Equilibrium solubility and solute-solvent interactions of carvedilol (Form I) in twelve mono solvents and its application for supercritical antisolvent precipitation. J. Mol. Liq..

[32] Ha E.-S., Kuk D.-H., Kim J.-S., Kim M.S. (2019). Solubility of trans-resveratrol in transcutol HP + water mixtures at different temperatures and its application to fabrication of nanosuspensions. J. Mol. Liq..

[33] Ha E.-S., Lee Y.-R., Kim M.-S. (2016). Solubility of dronedarone hydrochloride in six pure solvents at the range of 298.15 to 323.15K. J. Mol. Liq..

[34] Ha E.-S., Lee S.-K., Jeong J.-S., Sim W.-Y., Yang J.-I., Kim J.-S., Kim M.-S. (2019). Solvent effect and solubility modeling of rebamipide in twelve solvents at different temperatures. J. Mol. Liq..

[35] Ha E.-S., Choo G.-H., Baek I.-H., Kim M.-S. (2014). Formulation, characterization, and in vivo evaluation of celecoxib-PVP solid dispersion nanoparticles using supercritical antisolvent process. Molecules.

[36] Kim M.-S., Ha E.-S., Kim J.-S., Baek I., Yoo J.-W., Jung Y., Moon H.R. (2015). Development of megestrol acetate solid dispersion nanoparticles for enhanced oral delivery by using a supercritical antisolvent process. Drug Des. Devel. Ther..

[37] Ha E.-S., Choo G.-H., Baek I.-H., Kim J.-S., Cho W., Jung Y.S., Jin S.-E., Hwang S.-J., Kim M.-S. (2015). Dissolution and bioavailability of lercanidipine-hydroxypropylmethyl cellulose nanoparticles with surfactant. Int. J. Biol. Macromol..

[38] Ramos C. (2013). Development and validation of a headspace gas chromatographic method for determination of residual solvents in five drug substances. Int. J. Pharm. Sci..

[39] Das S., Lin H.S., Ho P.C., Ng K.Y. (2008). The impact of aqueous solubility and dose on the pharmacokinetic profiles of resveratrol. Pharm Res..

[40] Kapetanovic I.M., Muzzio M., Huang Z., Thompson T.N., McCormick D.L. (2011). Pharmacokinetics, oral bioavailability, and metabolic profile of resveratrol and its dimethylether analog, pterostilbene, in rats. Cancer Chemother. Pharmacol..

[41] Das S., Ng K.Y. (2011). Quantification of trans-resveratrol in rat plasma by a simple and sensitive high performance liquid chromatography method and its application in pre-clinical study. J. Liq. Chromatogr. Relat. Technol..

[42] Patil S.C., Tagalpallewar A.A.l, Kokare C.R. (2019). Natural anti-proliferative agent loaded self-microemulsifying nanoparticles for potential therapy in oral squamous. J. Pharm. Investig..

[43] Naik J.B., Waghulde M.R. (2018). Development of vildagliptin loaded Eudragit^®^ microspheres by screening design: In vitro evaluation. J. Pharm. Investig..

[44] De Marco I., Rossmann M., Prosapio V., Reverchon E., Braeuer A. (2015). Control of particle size, at micrometric and nanometric range, using supercritical antisolvent precipitation from solvent mixtures: Application to PVP. Chem. Eng. J..

[45] De Marco I., Prosapio V., Cice F., Reverchon E. (2013). Use of solvent mixtures in supercritical antisolvent process to modify precipitates morphology: Cellulose acetate microparticles. J. Supercrit. Fluids.

[46] Zupančič Š., Lavrič Z., Kristl J. (2015). Stability and solubility of trans-resveratrol are strongly influenced by pH and temperature. Euro. J. Pharm. Biopharm..

[47] Baek I., Kim J.-S., Ha E.-S., Choo G.-H., Cho W., Hwamg S.-J., Kim M.-S. (2014). Dissolution and oral absorption of pranlukast nanosuspensions stabilized by hydroxypropylmethyl cellulose. Int. J. Biol. Macromol..

[48] Maier-Salamon A., Hagenauer B., Wirth M., Gabor F., Szekeres T., Jager W. (2006). Increased transport of resveratrol across monolayers of the human intestinal Caco-2 cells is mediated by inhibition and saturation of metabolites. Pharm. Res..

[49] Planas J.M., Alfaras I., Colom H., Juan M.E. (2012). The bioavailability and distribution of trans-resveratrol are constrained by ABC transporters. Arch. Biochem. Biophys..

[50] Peñalva R., Morales J., González-Navarro C.J., Larrañeta E., Quincoces G., Peñuelas I., Irache J.M. (2018). Increased oral bioavailability of resveratrol by its encapsulation in casein nanoparticles. Int. J. Mol. Sci..

[51] Chang C.W., Wong C.Y., Wu Y.T., Hsu M.C. (2017). Development of a solid dispersion system for improving the oral bioavailability of resveratrol in rats. Eur. J. Drug Metab. Pharmacokinet..

