# Supplementary material for: Pure Trans-Resveratrol Nanoparticles Prepared by a Supercritical Antisolvent Process Using Alcohol and Dichloromethane Mixtures: Effect of Particle Size on Dissolution and Bioavailability in Rats
Source: Antioxidants (Basel). 2020 Apr 22;9(4):342. doi: 10.3390/antiox9040342 (PMC7222356; doi:10.3390/antiox9040342)
Supplement: Supplementary file 1 [file antioxidants-09-00342-s001.pdf]

antioxidants

MDPI

Table S1. DSC data of raw material, SAS processed nanoarticles and milled microparticles consisted of pure trans-resveratrol.

Samples		Melting peak temperature (°C)	Fusion enthalpy (ΔH, J/g)
Raw material		267.7 ± 0.7	265.6 ± 2.9
Nanoparticles (SAS processed, Solvent composition mass%)	MeOH/DCM=25/75	267.4 ± 0.6	263.7 ± 1.7
	MeOH/DCM=50/50	266.8 ± 0.7	261.5 ± 2.3
	MeOH/DCM=75/25	268.5 ± 0.7	259.7 ± 2.8
	MeOH/DCM=100/0	267.1 ± 0.6	264.3 ± 2.7
	EtOH/DCM=25/75	268.1 ± 0.6	260.2 ± 1.9
	EtOH /DCM=50/50	266.3 ± 0.5	258.8 ± 2.9
	EtOH /DCM=75/25	268.5 ± 0.6	262.6 ± 2.8
	EtOH /DCM=100/0	266.8 ± 0.8	263.1 ± 2.6
Microparticles (milled by Fitz mill)		268.1 ± 0.3	257.6 ± 5.3
Microparticles (milled by air jet-mill)		267.6 ± 0.5	261.7 ± 1.4

Data are expressed as mean ± standard deviation (n = 3). MeOH: methanol, DCM: dichloromethane, EtOH: ethanol.
